# Exposure to SARS-CoV-2 in Hospital Environment: Working in a COVID-19 Ward Is a Risk Factor for Infection

**DOI:** 10.3390/pathogens10091175

**Published:** 2021-09-12

**Authors:** Abeline Kapuczinski, Christophe de Terwangne, Steven De Keukeleire, Jean-Christophe Goffard, Antonio Sorgente, Sammy Place, Michael De Cubber

**Affiliations:** 1 Department of Internal Medicine, Centre Hospitalier EpiCURA, 7301 Hornu, Belgium; christophe_terwangne@hotmail.com (C.d.T.); sammy.place@epicura.be (S.P.); 2 Department of Laboratory Medicine, Centre Hospitalier EpiCURA, 7301 Hornu, Belgium; stevendekeukeleire@hotmail.com; 3 Department of Internal Medicine, Erasmus Hospital, Université Libre de Bruxelles, 1070 Bruxelles, Belgium; jean-christophe.goffard@erasme.ulb.ac.be; 4 Department of Cardiology, Centre Hospitalier EpiCURA Hospital, 7301 Hornu, Belgium; sorgente.antonio@gmail.com (A.S.); michael.decubber@epicura.be (M.D.C.)

**Keywords:** COVID-19, COVID-19 units, health care workers, infection, SARS-CoV-2

## Abstract

**Aims.** Health care workers (HCWs) are at risk of acquiring the Severe Acute Respiratory Syndrome Coronavirus 2 Infection (SARS-CoV-2). The aim of the study is to determine the SARS-CoV-2 positivity rates during the first epidemiologic peak among HCWs of a south Belgian hospital and to identify risks factors for infection. **Methods.** All hospital staff who worked during the first epidemiological peak were asked to answer a questionnaire regarding demographical data, function, type of working unit, type of contact with patients, eventual symptomatology, and the positivity of reverse transcription-polymerase chain reaction (RT-PCR) testing or immunoassay. **Results**. A total of 235 questionnaires were collected; 90 (38%) HCWs tested positive for SARS-CoV-2 from either RT-PCR or immunoassay testing. The positivity rate of HCWs between wards was statistically different (*p* = 0.004) and was higher in COVID-19 wards than Intensive Care Unit (ICU) and Emergency Department (ED). A total of 114 (49%) HCWs presented SARS-CoV-2-compatible symptomatology; 79 (88%) were positive on either RT-PCR or immunoassay testing; 74 (37%) HCWs were unable to work during the studied period; 5 were hospitalized. No deaths were reported. Multivariate logistic regression modeling showed that having symptoms was highly associated with test positivity (OR 23.3, CI 11.1, 53.1, *p*-value < 0.001). Working in a COVID-19 ward against working in ICU or ED was also predictive of positivity among HCWs (OR 3.25, CI 1.50, 7.28, *p*-value = 0.003). **Discussion and Conclusions.** This study shows a higher positivity rate compared to already reported positivity rates among HCWs. Reported differences in positivity rates depend on many factors, such as local crisis intensity, screening strategy, training in use of self-protective equipment, and study selection bias. HCWs working in COVID-19 wards, in comparison to ED and ICU, seemed at greater risk of being infected in this study. This could be explained by the disparity of HCWs’ experience in handling self-protective equipment and knowledge in infection prevention. Hence, care should be taken in proper training for less-experienced HCWs during hospital epidemics. The latter could increase HCWs’ protection and consequently decrease work absenteeism, ensuring enhanced continuity of patient care during hospital crisis. Rapid quarantine of symptomatic HCWs could reduce contamination rates, as having symptoms was highly associated with test positivity in this study.

## 1. Introduction

Health care workers (HCWs) and other hospital staff are at increased risk of acquiring the Severe Acute Respiratory Syndrome Coronavirus 2 Infection (SARS-CoV-2) [1]. Hence, highly transmissible viral epidemics such as the coronavirus disease 2019 (COVID-19) crisis put hospitals and hospital staff under enormous stress. Different hospital strategies had to be rapidly put in place to optimize infection prevention among HCWs and uninfected inpatients. These strategies were directed and implemented by an improvised reinforced staff specialized in hospital infection control.

To prevent infection spreading among hospitalized patients and among HCWs, at EpiCURA Hospital, wards were rapidly reorganized into COVID-19 wards according to influx of COVID-19 patients. Due to canceled hospital activities (elective surgery, consultations, etc.) HCWs were distributed to enforce the COVID-19 wards or other hospital activities.

In the early phase of the March to May 2020 first epidemiological crisis, and due to scarcity of laboratory resources, reverse transcription-polymerase chain reaction (RT-PCR) testing for screening COVID-19 among HCWs was limited to symptomatic patients, as well as a minority of hospital staff. During the month of April 2020, the first immunoassays with sufficient sensitivity and specificity were developed to assess for presence of SARS-CoV-2-directed immunoglobulins (IgG) in previously exposed or infected patients [2].

The previously reported SARS-CoV-2 positivity rates among HCWs vary widely between 1.6% and 33% [3,4,5,6,7].

This study aims to determine the SARS-CoV-2 exposure rate among HCWs at EpiCURA Hospital, through either RT-PCR positivity or immunoassay positivity, and to identify potential factors that could influence infection among HCWs.

## 2. Results

Table 1 shows results from the 235 collected questionnaires that were eligible for analysis.

A total of 90 (38%) HCWs tested positive for SARS-CoV-2, from either RT-PCR or immunoassay testing; 76 (32%) HCWs underwent a RT-PCR test on a nasopharyngeal swab during the epidemic; 47 (52%) were positive. In May 2020, when immunoassays were available, 226 (96%) HCWs benefited from immunoassay testing with a positivity rate of 38.5%. All RT-PCR-positive HCWs developed immunoglobulins.

The most-represented HCWs were Nurses with 166 (71%) collected questionnaires, followed by Paramedicals with 26 (11%), and Technicians with 24 (10%). Among the 235 HCWs, 158 (67%) worked mainly in COVID-19 wards and 77 (33%) in Emergency Department (ED) or in Intensive Care Unit (ICU). The positivity rate of HCWs between these wards is statistically different (*p* = 0.004). There was no difference in positivity rates between the different age classes and different HCWs functions.

**Figure 1:**
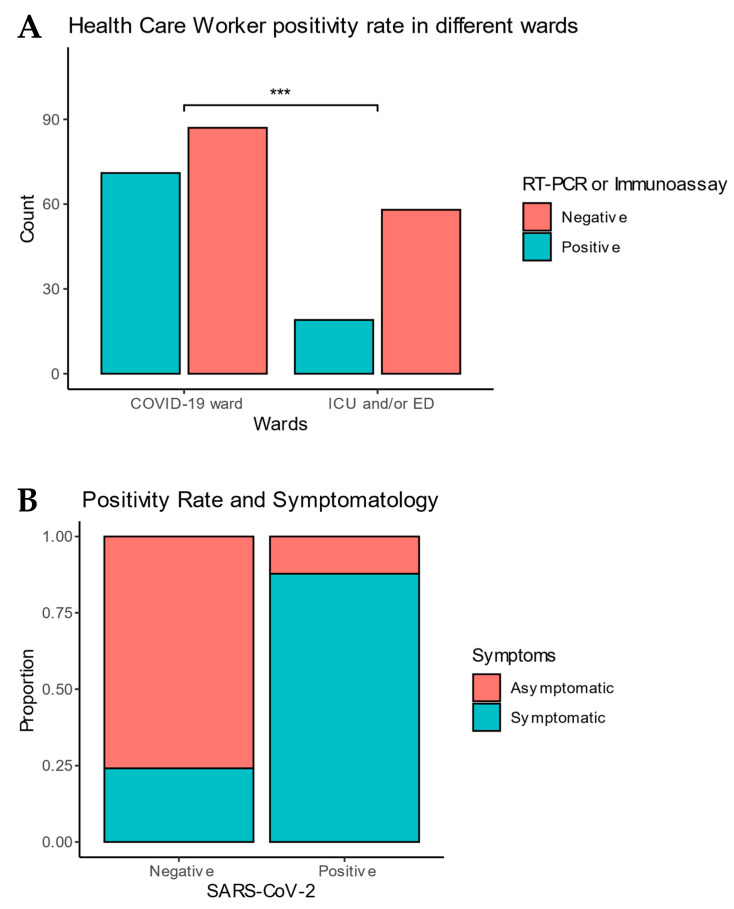
(**A**) Health care workers positivity in function of ward; (**B**) Health Care Workers positivity in function of symptomatology. *** defines statistical significant difference at *p* < 0.05.

The most frequent symptoms seemed to be flu-like symptoms (40%); ear, nose and throat (ENT) symptoms (31%), which included loss of taste, anosmia, dizziness, and sore throat; and respiratory symptoms (26%). Among the 114 HCWs who presented SARS-CoV-2-compatible symptoms, 79 (88%) were positive on either RT-PCR or immunoassay testing (Figure 1).

A total of 142 (61%) subjects claimed to have respected social distancing at work and 217 (93%) confirmed that they respected containment measures outside the hospital according to the government’s rules during the epidemiologic peak.

A total of 52 subjects (22%) were vaccinated against influenza. Among them, 30 (21%) tested negative for COVID-19 and 22 (24%) tested positive (*p* = 0.6). The amount of symptomatic flu-vaccinated SARS-CoV-2-positive HCWs was not different in comparison with the amount of symptomatic non-vaccinated SARS-CoV-2-positive HCWs (*p* = 0.8).

A total of 74 (37%) HCWs were incapacitated during the epidemic due to sickness during the studied period; 5 HCWs were hospitalized, but we did not report any deaths at our hospital.

Multivariate logistic regression modeling showed firstly that having symptoms was highly associated with test positivity (OR 23.3, CI 11.1, 53.1, *p*-value < 0.001). Working in a COVID-19 ward against working in ICU or ED was also predictive of positivity among HCWs (OR 3.25, CI 1.50, 7.28, *p*-value = 0.003) (Table 2). Finally, in multivariate modeling, exposure to high-risk contacts lost statistical significance (OR 3.08, CI 0.89, 11.9, *p*-value = 0.085).

## 3. Discussion

This study describes SARS-CoV-2 positivity rate among HCWs active during the epidemiological crisis and implied in patient care during that period. Furthermore, this study provides insights on infection risk factors among HCWs. 

Overall, 90 (38%) out of 235 HCWs tested positive on either RT-PCR or immunoassay testing.

The positivity rate of our study was firstly compared to the positivity rate of 24.9% (406/1630) calculated on the total number of hospital staff tested by immunoassays at EpiCURA Hospital laboratory during the period of May to October 2020. The difference between the positivity rate of our study and the total positivity rate of the hospital can be explained because the latter reflects a raw number of tests. Indeed, these included HCWs and hospital staff who were not working during the period of March to May 2020 (epidemiological peak) and did also included those who were not directly involved in patient care (kitchen, administration, etc.). Moreover, there is an important refusal bias, as only a few HCWs who were not infected answered the questionnaire.

The positivity rate of SARS-CoV-2 was also higher compared to other national and international reports (Table 3, complementary data).

In Belgium, Martin et al. reported a SARS-CoV-2 positivity rate of 12.6% at the Centre Hospitalier Universitaire Saint-Pierre, detected by RT-PCR and immunoassay [3]. This report included a population of HCWs working exclusively in COVID-19 wards but was nevertheless lower than the present report. A public hospital in the center of Brussels reported a rate of 14.6% by analyzing all hospital staff, regardless of function or status [4].

In China, Chen et al. studied the prevalence of SARS-CoV-2 among HCWs directly exposed to COVID-19-confirmed patients and reported a rate of 17.1% of seroconversion by immunoassay testing [17]. In Italy, a study showed a positivity rate of 9% among HCWs of an entire otolaryngology unit, using RT-PCR tests in the presence of symptoms and immunoassay tests in the absence of symptoms [5]. Another study in Germany demonstrated a very low seroconversion rate of 1.6%, tested by immunoassay only in wards with COVID-19-confirmed patients [6]. In the United States (New York City), Mansour et al. showed a seroconversion rate of 36%, which is similar to our study, using immunoassays only among HCWs with direct physical contact, and aerosol procedures in ED and ICU or in HCWs exposed directly to patients [7]. Hence, the SARS-CoV-2 positivity rate among HCWs varies widely between 1.6% and 33% [3,4,5,6,7] (Table 3). The reported positivity rate depends on many factors. Firstly, important selection bias is found through analysis of the different reports. Some studies report positivity rates of total hospital staff [4], HCWs of a single unit [5], HCWs of COVID-19 wards, or HCWs in contact with infected patients and/or HCWs at high risk of infection [3,6]. Moreover, different immunoassays were used throughout different studies. Secondly, positivity rates could be influenced by local crisis intensity, which could be influenced by local lockdown rules and local strategies of epidemic disease control and screening. These factors could highly impact preparation time for re-organizing adequate hospital activity. Lastly, positivity rates can depend on training intensity of HCWs in the handling of self-protective equipment along with availability of equipment. For these reasons, care should be taken in comparing different positivity rates of HCWs throughout different countries. Reports of infection rates of the first epidemiological peak are of utmost importance in the understanding of in-hospital exposure, as future SARS-CoV-2 incidence calculations through seroconversion could be biased by previous exposure.

Among the HCWs in our hospital who were tested by RT-PCR during the crisis, 52% were positive. This could be explained by the scarcity of resources and PCR kits and because only symptomatic HCWs were tested during the first epidemiological peak.

This study highlighted several risks factors for HCWs acquiring COVID-19. We observed a difference between the positivity rate of HCWs in COVID-19 wards and ICU/ED wards. It seemed that HCWs working in a COVID-19 ward had greater odds of being positive compared to HCWs working in ICU or ED. This could be due to the more advanced experience in self-protection protocols and equipment of HCWs in acute medical settings such as ICU or ED. However, a recent review showed that HCWs working in ICU may have an increased risk of infection because of adverse effects due to personal protective equipment [18]. During the re-organization of hospital activity and preparation in response to the crisis, some nurses and caregivers were recruited from revalidation wards or outpatient clinics to help in hospital care, with express training in self-protective equipment. Thus, a disparity in HCWs’ experience in handling of self-protective equipment and infection prevention could have influenced positivity rates and may explain lower rates in ICU and ED in our study. Care should be taken in proper training of less experienced HCWs during hospital epidemics. This could increase HCWs’ protection and even reduce anxiety among HCWs, which consequently could decrease work absenteeism and ensure enhanced continuity of patient care during hospital crises.

Another explanation is work organization on each ward. Indeed, HCWs in ED/ICU worked according to a 12 h shift, whereas HCWs in COVID-19 wards worked in 8 h shift, increasing the number of working HCWs and contacts between them during debriefing and lunch breaks.

Exposure to high-risk contacts, however, was not significant after multivariate adjustment of risk factors.

Our study did not show any effects of age, function in hospital, contact frequency, or duration (in days) of working in a COVID-19 ward during the first epidemiological peak.

The impact of absenteeism was significant (37%) and challenged the maintenance of proper hospital activity. From the 90 positive subjects, only 5 were hospitalized, and there was zero reported death. Most were mild diseases. Interestingly, there was no difference in positivity rates according to different age classes.

Among the population studied in this report, 52 (22%) had been flu-vaccinated. Despite a study reporting the protective role of the influenza vaccine on COVID-19 incidence and its contribution to the COVID-19-related burden on the healthcare system [19,20,21], we did not find any association between flu-vaccinated HCWs and non-vaccinated HCWs and development of COVID-19-related symptoms among SARS-CoV-2-positive HCWs.

The main limitation of this study was the survey-based, single-center retrospective study design. Moreover, there is a lack of knowledge of the underlying health conditions of all HCWs. The surveys were based on self-reported symptoms, which could have led to an over-reporting of symptoms.

## 4. Materials and Methods

This is a single-center, multisite, retrospective analysis carried out at Centre Hospitalier EpiCURA, a secondary care center in the south of Belgium. The study was conducted in two of the three sites of EpiCURA, which account for 569 out of 806 hospital beds. These two sites employ a total of 1916 employees during normal hospital activity. The number of employees was significantly reduced during crisis to the most essential HCWs.

**Figure 2:**
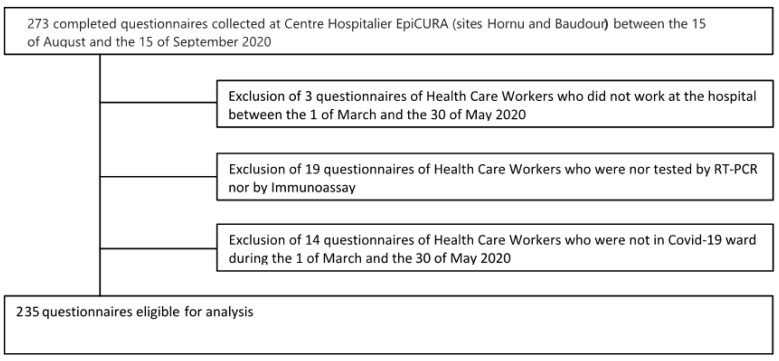
Flowchart of analyzed questionnaires.

During the month of August 2020, all staff who worked between the 1st of March 2020 and the 30th of May 2020 at EpiCURA (site Hornu and site Baudour) were asked to complete an anonymized questionnaire regarding demographic data, function in hospital, type of working unit, type of contact with patients, eventual symptomatology, and the positivity of RT-PCR testing or immunoassay. Questionnaires were put in different wards and participants were freely asked to complete them. A total of 273 completed questionnaires were collected on 15 September 2020. We included for analysis all completely filled-in questionnaires of employees and HCWs that were either tested by RT-PCR or by immunoassay. Among these 273 questionnaires, 38 were excluded from analysis because they were filled out by those who had worked outside the defined dates, were not tested by either RT-PCR or immunoassay, or did not work in a COVID-19 ward during the defined dates (Figure 2).

HCWs functions were organized into four different groups: *Nurse*, including nurses and caregivers; *Paramedical*, including physiotherapists, speech therapists, dietitians, occupational therapists, psychologists, and medical imagery technologists; *Physician*; and lastly, *Technician*, including social workers, cleaning operative, secretaries, administration, and stretcher bearers.

Wards were organized as COVID-19 wards, emergency department (ED), intensive care unit (ICU), and COVID-19-free wards, because hospital organization was reduced to emergencies and COVID-19 activities, in accordance with Belgium’s rules during the pandemic.

The type of contact of HCWs with a SARS-CoV-2-affected patient was defined as *low risk* if there was no physical contact, respect of social distancing (more than 1.5 m between HCW and patient), and a duration of less than 15 min; and *high risk* in the case of physical contact through care and/or aerosol treatments, several times a day, for more than 15 min with a COVID-19-confirmed patient.

All HCWs had FFP2 masks, gloves, and protective suits in case of contact with a COVID-19 patient. To protect for potential ocular transmission, HCWs used visors [22]. Moreover, HCWs wore surgical masks at all times, in accordance with Belgian guidelines. In ED, HCWs had to use self-protection in all circumstances; the same was true for ICU, where almost all the patients were COVID-19 positive. Adherence to hygiene measures was checked three times a week by hygienists and infectious diseases specialists within the hospital.

The immunoassay used was the LIAISON^®^SARS-CoV-2 from DiaSorin^®^, a chemiluminescence immunoassay (CLIA), allowing the detection of IgG antibodies specifically directed against SARS-CoV-2, which was validated in Belgium. This immunoassay has an excellent analytical and clinical performance [23] and demonstrated a sensitivity of 97.4% at 15 days post-confirmed PCR and a specificity of 98.9% [2]. The given result is negative under 12.0 AU/mL, equivocal between 12.0 and 15.0 AU/mL, and positive over 15.0 AU/mL. The RT-PCR method used was an extraction by Qiacube (Qiagen) and STARlet (Seegene), followed by amplification by Roto-Gene Q MDX (Qiagen) and CFX96 (Bio-Rad). A cycle threshold below 40 is used as cut-off for positivity rate.

As RT-PCR testing was performed only in the case of symptoms during the first epidemiological peak, HCWs tested with positive immunoassays during June and July were added to have a closer approximation of contamination of HCWs during the studied period.

The SARS-CoV-2 positivity rate among HCWs at EpiCURA Hospital during the first epidemiological peak of the pandemic was compared to the positivity rates during the first peak at other hospitals in Belgium and in other countries. A literature review was carried out between October 2020 and December 2020 (complementary data).

The study was approved by the local Ethics Committee of EpiCURA Hospital with the reference P2020/032 on 19th August 2020 and was performed in accordance with the ethical standards of the 1964 Declaration of Helsinki and its later amendments.

### Statistical Analysis

Categorical variables were expressed as count (percentage), and continuous variables as median (25–75th percentiles), as appropriate. Differences between groups were assessed using a chi-square test with Yates continuity correction or the Fischer exact test for categorical variables, as appropriate, and Kruskal–Wallis or Mann–Whitney U test for continuous variables. To assess for predictive factors, multivariate binomial logistic regression was used with previously statistically significant variables (contact risk, wards, and symptoms). A threshold *p*-value of 0.05 was used to define statistically significant tests. Statistical analyses were performed using R: A Language and Environment for Statistical Computing, R Core Team, Vienna, Austria, 2020.

## 5. Conclusions

This study shows, despite some limitations, a high positivity rate compared to reported positivity rates among HCWs. Reported positivity rates depend on many factors, such as local crisis intensity, screening strategy, training in use of self-protective equipment, and study selection bias. Hypothetically, disparity of experience among HCWs in handling of self-protective equipment and infection prevention could have influenced positivity rates and may explain lower infection rates in the ICU and ED in our study.

Rapid quarantine of symptomatic HCWs could reduce contamination rates, as having symptoms was highly associated with test positivity in this study.

## Figures and Tables

**Table 1:** General characteristics of health care workers in function of positivity of either reverse transcription-polymerase chain reaction (RT-PCR) or immunoassay testing.

	Overall, N = 235	PCR or Immunoassay		*p*-Value ^2^
		Negative, N = 145 ^1^	Positive, N = 90 ^1^	
**Function**				0.3
Nurse	166 (71%)	98 (68%)	68 (76%)	
Paramedical	26 (11%)	17 (12%)	9 (10%)	
Physician	19 (8.1%)	11 (7.6%)	8 (8.9%)	
Technician	24 (10%)	19 (13%)	5 (5.6%)	
**Age class**				0.3
<29 years	64 (27%)	40 (28%)	24 (27%)	
>60 years	6 (2.6%)	5 (3.4%)	1 (1.1%)	
30–39 years	72 (31%)	43 (30%)	29 (32%)	
40–49 years	53 (23%)	37 (26%)	16 (18%)	
50–59 years	40 (17%)	20 (14%)	20 (22%)	
**BMI**	24.3 (21.5, 27.8)	24.2 (21.6, 27.8)	24.7 (21.5, 28.0)	0.6
**Sex**				0.012
Female	182 (77%)	104 (72%)	78 (87%)	
Male	53 (23%)	41 (28%)	12 (13%)	
**Ward**				0.004
COVID-19 ward	158 (67%)	87 (60%)	71 (79%)	
ICU and/or ED	77 (33%)	58 (40%)	19 (21%)	
**Contact risk**				0.038
High Risk	195 (88%)	112 (84%)	83 (94%)	
Low Risk	26 (12%)	21 (16%)	5 (5.7%)	
**Respect of social distancing at work**				0.6
Not respected	11 (4.7%)	7 (4.8%)	4 (4.5%)	
Partly respected	80 (34%)	46 (32%)	34 (39%)	
Well respected	142 (61%)	92 (63%)	50 (57%)	
**Respect of social distancing outside work**				0.5
Not respected	2 (0.9%)	2 (1.4%)	0 (0%)	
Partly respected	14 (6.0%)	10 (7.0%)	4 (4.4%)	
Well respected	217 (93%)	131 (92%)	86 (96%)	
**Symptoms**	114 (49%)	35 (24%)	79 (88%)	<0.001
Fever	51 (22%)	13 (9.0%)	38 (42%)	<0.001
Dyspnea	38 (16%)	10 (6.9%)	28 (31%)	<0.001
Flu-like symptoms	93 (40%)	27 (19%)	66 (73%)	<0.001
Respiratory symptoms	62 (26%)	16 (11%)	46 (51%)	<0.001
ENT ^3^ symptoms	72 (31%)	10 (6.9%)	62 (69%)	<0.001
Abdominal discomfort	43 (18%)	11 (7.6%)	32 (36%)	<0.001
Skin lesions	10 (4.3%)	3 (2.1%)	7 (7.8%)	0.047
**Influenza vaccination 2019**	52 (22%)	30 (21%)	22 (24%)	0.6
**Thorax Tomodensitometry**	20 (8.5%)	7 (4.8%)	13 (14%)	0.020
Pulmonary lesions on TDM	10 (50%)	0 (0%)	10 (77%)	0.003
**qRT-PCR testing**	76 (32%)	29 (20%)	47 (52%)	NA
**Immunoassay testing**	226 (96%)	139 (96%)	87 (97%)	NA
**Hospitalization**	5 (4.5%)	0 (0%)	5 (6.2%)	0.3
**Work absenteeism**	74 (37%)	17 (15%)	57 (66%)	<0.001

^1^ Statistics presented: n (%); median (IQR). ^2^ Statistical tests performed: chi-square test of independence; Fisher’s exact test; Wilcoxon rank-sum test. ^3^ ENT: Ear, nose, and throat.

**Table 2:** Multivariate binomial logistic regression of SARS-CoV-2 positivity predictors.

	OR ^1^	95% CI ^1^	*p*-Value
**Symptoms**			
Asymptomatic	—	—	
Symptomatic	23.3	11.1, 53.1	<0.001
**Risk**			
Low Risk (reference level)	—	—	
High Risk	3.08	0.89, 11.9	0.085
**Ward**			
ICU and/or ED (reference level)	—	—	
COVID-19 ward	3.25	1.50, 7.28	0.003

^1^ OR = odds ratio, CI = confidence interval.

**Table 3:** Complementary data: reported positivity rates in different countries and hospitals.

Author Hospital Country	Hospital and Country	Studied Population	Detection Method	Reported Positivity Rate	Additional Findings
Martin et al. [3]	Saint Pierre Hospital Belgium	N = 326 HCWs from COVID-19, ED, ICU wards	RT-PCR and Immunoassay	12.6%	Screening of all groups of HCWs in highly exposed COVID-19 units is recommended.
Blairon et al. [4]	Reseau IRIS Belgium	N = 3145 hospital staff from COVID-19 and COVID-19 free wards	RT-PCR and Immunoassay	14.6%	
Paderno et al. [5]	Italy (Northern Italy)	N = 58 from a otolaryngology unit	RT-PCR if symptoms and Immunoassay if absence of symptoms	9%	The prevalent risk of infection was related to extrahospital contacts
Sotgiu et al. [8]	Italy (Milan)	N = 202 HCWs from COVID-19 wards	Immunoassay (IgM, IgG)	IgM: 14.4% IgG: 7.41%	IgM are higher in the age group 20–29 and 60–69
Lahner et al. [9]	Italy (Rome)	N = 2057 HCWs from a COVID-19 regional hub during the pandemic	RT-PCR and Immunoassay (IgM, IgG)	RT-PCR: 2.7% IgM: 0% IgG: 0.7%	Seroprevalence is higher in HCW than general population; IgM seems not to be useful test for active Sars-Cov-2infection
Korth et al. [6]	Germany	N = 316: HCWs with direct contact with COVID-19 confirmed patients	Immunoassay	1.6%	Good local hygiene standard
Lackermair et al. [10]	Germany	N = 151 HCWs	Immunoassay	2.6%	
Kasper Iversen et al. [11]	Denmark (Capital region)	N = 29117 HCWs including students	Immunoassay (IgM, IgG)	4.04%	Comparison with a blood donor group with positivity rate was inferior
Sikkema et al. [12]	Netherlands (from 9 hospitals)	N = 1796 symptomatic HCWs	RT-PCR	5%	
Garcia-Basteiro et al. [13]	Spain	N = 578 HCWs from COVID-19 wards and COVID-19-free wards	Immunoassay (IgM, IgG, IgA)	9.3%	IgA demonstrating the highest sensitivity in the initial days after symptoms onset
Mansour et al. [7]	USA (NYC)	N = 285 HCWs with high risk with contacts and aerosols	Immunoassay	36%	
Jeremias et al. [14]	USA (NYC)	N = 3046 HCWs	RT-PCR if symptoms and Immunoassay without symptoms	9.8%	PPE confers protection and lower infection rates of COVID-19 among HCWs
Barret et al. [15]	USA	N = 546 HCWs and N = 283 non HCWs	RT-PCR	HCWs: 7.3% Non HCWs: 0.4%	Comparison HCWs with non-HCWs
Vahidy et al. [16]	USA (Texas)	N = 2787 HCWs and non-clinical workers from COVID-19 wards and COVID-19-free wards	RT-PCR	Total: 3.9% COVID-19 wards: 5.4%; COVID-19-free wards: 0.6%	
Chen et al. [17]	China	N = 105 HCWs exposed to confirmed COVID-19 patients	Immunoassays	17.1%	

## Data Availability

Data can be asked on demand to the authors.
